# Pathogenic role of Twist-1 protein in hydatidiform molar pregnancies and investigation of its potential diagnostic utility in complete moles

**DOI:** 10.1186/s13000-023-01329-5

**Published:** 2023-03-29

**Authors:** Behnaz Jahanbin, Soheila Sarmadi, Dorsa Ghasemi, Fatemeh Nili, Jafar-Ali Moradi, Soha Ghasemi

**Affiliations:** 1 Department of Pathology, Cancer Institute, Imam Khomeini Hospital Complex, Tehran University of Medical Sciences, End of Keshavarz Ave, , Tehran, IR Iran; 2 Department of Pathology, Yas Women Hospital, Tehran University of Medical Sciences, Tehran, IR Iran; 3 Qazvin University of Medical Sciences, Tehran, IR Iran

**Keywords:** Hydatidiform mole, Complete mole, Partial mole, Diagnosis, Immunohistochemistry, Twist-1

## Abstract

**Background:**

Complete and partial moles (PM) are the most common gestational trophoblastic diseases. Due to some overlapping morphological findings, ancillary studies may be necessary.

**Methods:**

In this cross-sectional study, 47 cases of complete mole (CM) and 40 cases of PM were randomly selected based on histopathological criteria. Only those cases that were agreed upon by two expert gynecological pathologists and confirmed by the P57 IHC study were included. The expression level of the Twist-1 marker in villi stromal cells, as well as syncytiotrophoblasts, was evaluated quantitatively (percentage of positive cells), qualitatively (staining intensity) and as a total comprehensive score.

**Results:**

Expression of Twist-1 is higher and more intense in villous stromal cells of CMs (p < 0.001). Moderate to strong staining intensity in more than 50% of villous stromal cells, can differentiate CM and PM with 89.5% sensitivity and 75% specificity. In syncytiotrophoblasts of CM, Twist-1 expression was significantly lower than PM (p < 0.001). Negative or weak staining intensity in less than 10% of syncytiotrophoblasts, can distinguish CM and PM with 82.9% sensitivity and 60% specificity.

**Conclusion:**

A higher expression of Twist-1 in villous stromal cells of hydatidiform moles is a sensitive and specific marker for the diagnosis of CMs. An elevated expression of this marker in villous stromal cells suggests another pathogenic mechanism for more aggressiveness of CMs in addition to the characteristics of trophoblast cells. The opposite result was obtained in the expression of Twist-1 in the syncytiotrophoblasts, compatible with defects in the process of formation of these supportive cells in CMs.

## Background

Gestational trophoblastic disease encompasses a spectrum of pregnancy-related disorders, ranging from premalignant disorders of complete and partial hydatidiform mole, and the malignant disorders of invasive mole, choriocarcinoma, and the rare placental-site trophoblastic tumor [1, 2].

Hydatidiform mole (HM) refers to an abnormal pregnancy characterized by varying degrees of trophoblastic proliferation (both cytotrophoblasts and syncytiotrophoblasts) and vesicular swelling of placental villi associated with an absent or abnormal fetus/embryo. Its incidence is mainly affected by geographical location. South-eastern Asia, the Middle East, and South America show the highest cases, whereas it is the lowest in Western Europe and North America [1, 3]. Two syndromes of HM have been described based on both morphologic and cytogenetic criteria. Complete hydatidiform mole is the category without an embryo/fetal tissue exhibiting diandric diploid karyotype [4]. Partial hydatidiform moles on the other hand can demonstrate evidence of fetal development with a diandric triploid karyotype [4].

Clinical symptoms of a molar pregnancy generally include vaginal bleeding, large uterine size, severe vomiting, premature pre-eclampsia, absent fetal heartbeat, and a significant increase in serum ß-hCG levels. CMs previously presented in the late first or early second trimester with characteristic snowstorm-like ultrasonography. However, today, symptomatic women with vaginal bleeding are more likely to refer for abortion in the early stages of pregnancy due to the widespread use of sonography and quantitative measurement of ß-hCG that allow clinicians to make an earlier diagnosis, with most evacuated at the gestational age of 8 to 12 weeks. Partial moles (PM) are mostly clinically presented as missed abortions with a small uterus [5]. Sonography findings in the first trimester of molar pregnancy are less clear, with fair to moderate interobserver agreement [6].

Histopathological examination remains the cornerstone of the diagnosis of HM. CM is known by hydropic villi with cistern formation, trophoblastic proliferation with abnormal distribution, and loss of polarity [1, 7]. PM microscopic diagnosis is based on the identification of a mixture of two villous populations including small fibrotic and enlarged irregularly shaped villi with mild to moderate circumferential trophoblastic proliferation [7]. However, the diagnosis and classification of HM have become increasingly difficult because HMs are now commonly evacuated at an earlier stage and do not satisfy the well-established classic morphological features. The diagnosis of HM based on morphology alone is susceptible to inter-observer variability and therefore suboptimal diagnostic reproducibility [8, 9]. Differentiating a molar pregnancy from non-molar specimens (NMS) and the classification of HM as CM (including early CM), PM, or hydropic miscarriage is important for clinical practice and the outcome [5]; trophoblastic neoplasia (invasive mole or choriocarcinoma) follows CM in 15–20% of cases [1]; while less than 5% of PMs will develop postmolar gestational trophoblastic neoplasia (GTN); metastases occur rarely and the diagnosis of choriocarcinoma has not been confirmed after a PM [1].

The histological, clinical, and ultrasonographical manifestations in different molar gestations usually overlap, leading to a demanding process of making a final diagnosis. It is crucial to investigate new biomarkers and molecular techniques in clinical trials to establish a better routine practice of differentiating these subtypes of molar gestation [10].

The immunohistochemical study can play an important role in diagnosis. P57 (a paternally imprinted, maternally expressed gene), if absent can make a diagnosis in favor of CM, versus its positivity in hydropic abortions and PMs [11, 12]. Flow cytometry, cytogenetic study, and short tandem genomic imprinting can also assist in distinguishing diploid complete from triploid PMs [1, 13].

Twist-1 is an essential protein in epithelial-mesenchymal transition (EMT), especially in cancer formation and progression to invasive and metastatic tumors, and is notably expressed in carcinosarcomas. It is also required for trophoblastic differentiation, placental formation, gastrulation, mesoderm formation, and neural crest migration [14]. A recent study has shown that Twist-1 can be the marker of choice in the CM/PM differentiation [15].

## Methods

In this retrospective cross-sectional study, 87 cases were chosen from uterine curettage specimens with a diagnosis of molar pregnancy; 47 cases of CM and 40 cases of PM were randomly selected based on histopathological criteria, and by electronic search in the hospital information system (HIS) of pathology department of cancer institute of Imam Khomeini Hospital Complex (IKHC), Tehran, Iran from 2014 to 2017. Clinical characteristics of patients such as age, gestational age, the number of previous pregnancies, and serum ß-hCG level are determined based on patients’ clinical records. The study was approved by the local ethics committee of our university (IR.TUMS.IKHC.REC.1400.089).

Patients with the diagnosis of complete or partial mole which had been confirmed by P57 immunohistochemistry (IHC) entered the study; while the cases with insufficient and inappropriate pathology samples, cases with non-diagnostic IHC results, and the patients with unavailable clinical information were excluded. The cases were reviewed by two expert gynecopathologists (Dr. Soheila Sarmadi and Dr. Fatemeh Nili). Strict morphological criteria for the selection of HMs were applied. For the distinction of PM and CM, in addition to morphological findings, an IHC study for P57 was done.

After the selection of the appropriate block and preparing 3-*m*-thick unstained slides, overnight drying at 60 ºC, deparaffinization, rehydration, and heat-induced epitope retrieval were done. After blockage of endogenous peroxidase, the specimens were incubated with primary antibody P57 (MAD-000721 QD P57 9KP10, Master diagnostic: Spain) and Twist-1 (Mouse monoclonal antibody, 10E4E6, dilution: 1/100, Boston: USA) and finally Master Polymer Detection kit (HRP).

The p57 immunoreactivity was interpreted as satisfactorily negative when villous stromal cells and cytotrophoblasts were entirely negative or demonstrated only limited expression (nuclear staining < 10% of these cell types) with the presence of internal positive control (maternal decidua and/or intermediate trophoblastic cells exhibiting nuclear expression of p57). Positive p57 immunoreactivity was interpreted when the extent of staining in these cell types was extensive or diffuse.

Nuclear staining of Twist-1 in villous stromal cells and syncytiotrophoblasts was evaluated and analyzed as the following variables: the percentage of positive cells (PS), intensity of nuclear staining (IS): score 0 (no staining); score 33% (weak nuclear staining), score 66% (moderate nuclear staining) and score 100% (strong nuclear staining). A comprehensive score (CS) was calculated by multiplying IS and PS as described above.

Using Receiver-Operating Characteristic (ROC) curves, the best cut-off values for differentiation of molar pregnancies, the highest sensitivity, specificity, and positive and negative predictive values were calculated. The quantitative and qualitative variables were compared by Chi-square and independent sample T-tests, respectively. P-values less than 0.05 were considered significant.

## Results

### Demographic and clinical findings

87 women affected by molar pregnancy were included in the study. The mean age of the subjects was 28.22 ± 6.98 years ranged 17 to 52 years with a mean gestational age of 73.75 ± 13.66 days. The mean serum ß-hCG was 99935.84 ± 10,684,057 (median: 57,810, range: 1395-452614) IU/mL. Regarding gravidity, 38 (43.2%) were null gravid, 23 (26.1%) were primigravid and others were multigravid. Four patients (4.5%) had a previous history of molar pregnancy. Also, 26 (31.7%) expressed experiencing abortion. The mean age of patients and gestational age were not statistically different in CM and PM. Serum ß-hCG level was significantly higher in CM. Ultrasonographic examination diagnosed CMs with a higher prevalence in comparison to PMs. Past history of abortion and molar pregnancy was not significantly different in CM and PMs (Table 1).

**Table 1:** Clinical features of the cases with hydatidiform moles

Diagnosis	Partial mole	Complete mole	P value
Patients age (mean ± SD) Gestational age (day) ß-hCG level Ultrasonographic findings in favor of mole History of molar pregnancy History of abortion	28 ± 5.76 74.17 ± 14.00 147797.25 ± 121172.55 Yes: 10 No: 26 Yes: 1 No: 40 Yes: 11 No: 31	27.73 ± 7.35 72.71 ± 13.93 48197.88 ± 53182.56 Yes: 29 No: 9 Yes: 3 No: 41 Yes: 15 No: 29	0.36 0.56 < 0.001 < 0.001 0.384 0.379

### Twist-1 immunoreactivity in villous stromal cells

**Fig. 1:**
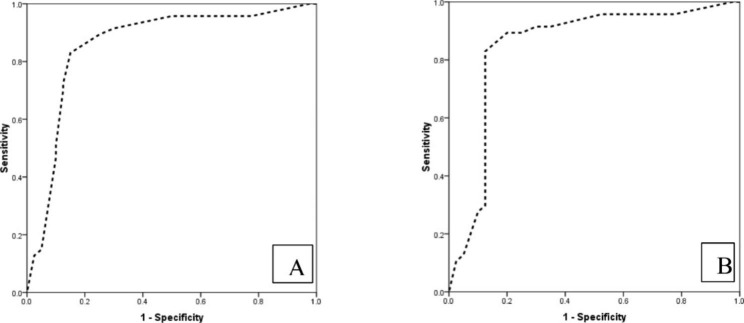
The ROC curve analysis in determining the value of percentage **(A)** and comprehensive score **(B)** of Twist-1 expression in villous stromal cells for the distinction of complete from partial molar pregnancy

The mean percentage of positive villous stromal cells in CM and PM pregnancies was 69.04 ± 18.98 and 32.75 ± 22.55, respectively (p < 0.001). The weak intensity of stromal cells staining was found in 10.6% and 75.0%, moderate in 59.6% and 12.5%, and strong in 29.8% and 12.5% of CM and PMs, respectively indicating a significantly higher intensity in CMs (p < 0.001). Similarly, the mean of stromal cell comprehensive score (CS) in complete and partial mole was 52.97 ± 23.81 and 19.16 ± 24.88, respectively which demonstrates a statistically significant difference (p < 0.001). According to ROC curve analysis, both percentages of stromal (AUC = 0.858, 95%CI: 0.770 to 0.946) and CS stromal cells (AUC = 0.846, 95% CI: 0.751 to 0.940) could differentiate complete from partial molar pregnancy (Fig. 1). Here, Twist-1 positive staining in more than 50% of stromal cells can differentiate CM and PM with 89.5% sensitivity, 75% specificity, 80.7% positive predictive value (PPV), and 85.7% negative predictive value (NPV) (Table 2).

**Table 2:** Diagnostic accuracy of Twist-1 expression in CM and PM in cut-off value 50% for villous stromal cells and 10% for syncytiotrophoblasts

	Complete mole	Partial mole	Sensitivity	Specificity	Positive predictive value	Negative predictive value
Stromal cells -Moderate to strong staining in > 50% of cells -Weak staining in < 50%	42 5	10 30	89.5%	75.0%	80.7%	85.7%
Syncytiotrophoblasts -Negative or weak staining in < 10% of cells -Moderate or strong staining in > 10% of cells	39 8	16 24	82.9%	60.0%	70.9%	75.0%

### Twist-1 immunoreactivity in syncytiotrophoblasts

**Fig. 2:**
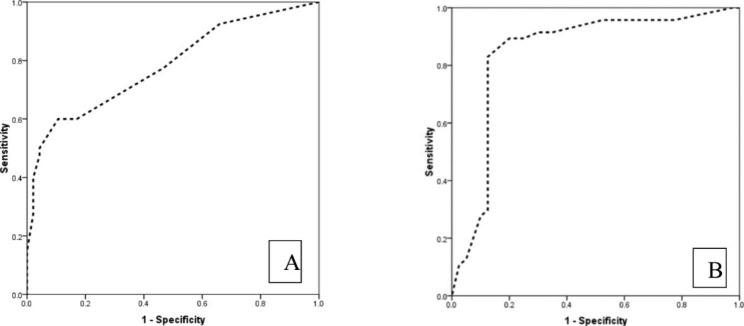
The ROC curve analysis in determining the value of percentage of Twist-1 expression in syncytiotrophoblasts **(A)** and CS **(B)** for the distinction of complete from partial molar pregnancy

The mean percentage of syncytiotrophoblast staining with Twist-1 in CM and PM pregnancies was 7.87 ± 9.31 and 27.75 ± 21.48, respectively (p < 0.001). Thirty-four (34%) of CMs and 7.5% of PMs were negative. A weak intensity in 59.6% and 42.5% and moderate intensity in 6.4% and 50.0% of CM and PMs were identified, respectively (p < 0.001). None of the cases reveal strong staining. Similarly, the mean CS was 3.11 ± 5.14 and 15.87 ± 14.91 in syncytiotrophoblasts of CM and PM, respectively (p < 0.001). According to ROC curve analysis, percentage (AUC = 0.782, 95%CI: 0.684 to 0.881) and CS of syncytiotrophoblast cells (AUC = 0.794, 95%CI: 0.697 to 0.891) could differentiate complete from partial molar pregnancy (Fig. 2). Negative or weak nuclear staining in less than 10% of syncytiotrophoblasts can differentiate CM and PM with 82.9% sensitivity, 60% specificity, 70.9% PPV, and 75% NPV (Table 2).

## Discussion

Complete and partial mole, as the most common types of GTDS, are genetically different disorders. Despite the different morphological findings, there are some overlapping histopathological features. The tumors are treated similarly, but the behavior is different with a higher probability of invasion in the CM. In this way, the patients with CMs are followed more rigorously than the patients with PMs.

The agreement between pathologists in differentiating the subtypes of molar pregnancies is fair [8, 9]. In some cases, the use of additional diagnostic tests such as IHC will be required. The high sensitivity and specificity of the P57 marker for differentiating complete and PMs have been confirmed in different studies [11, 12, 16]. However, based on the recent 2020 WHO classification of female genital tract tumors, molecular genotyping is essential to confirm the diagnosis of PMs [17]. Unfortunately, genetic testing of PM samples is expensive and not available in all centers.

**Fig. 3:**
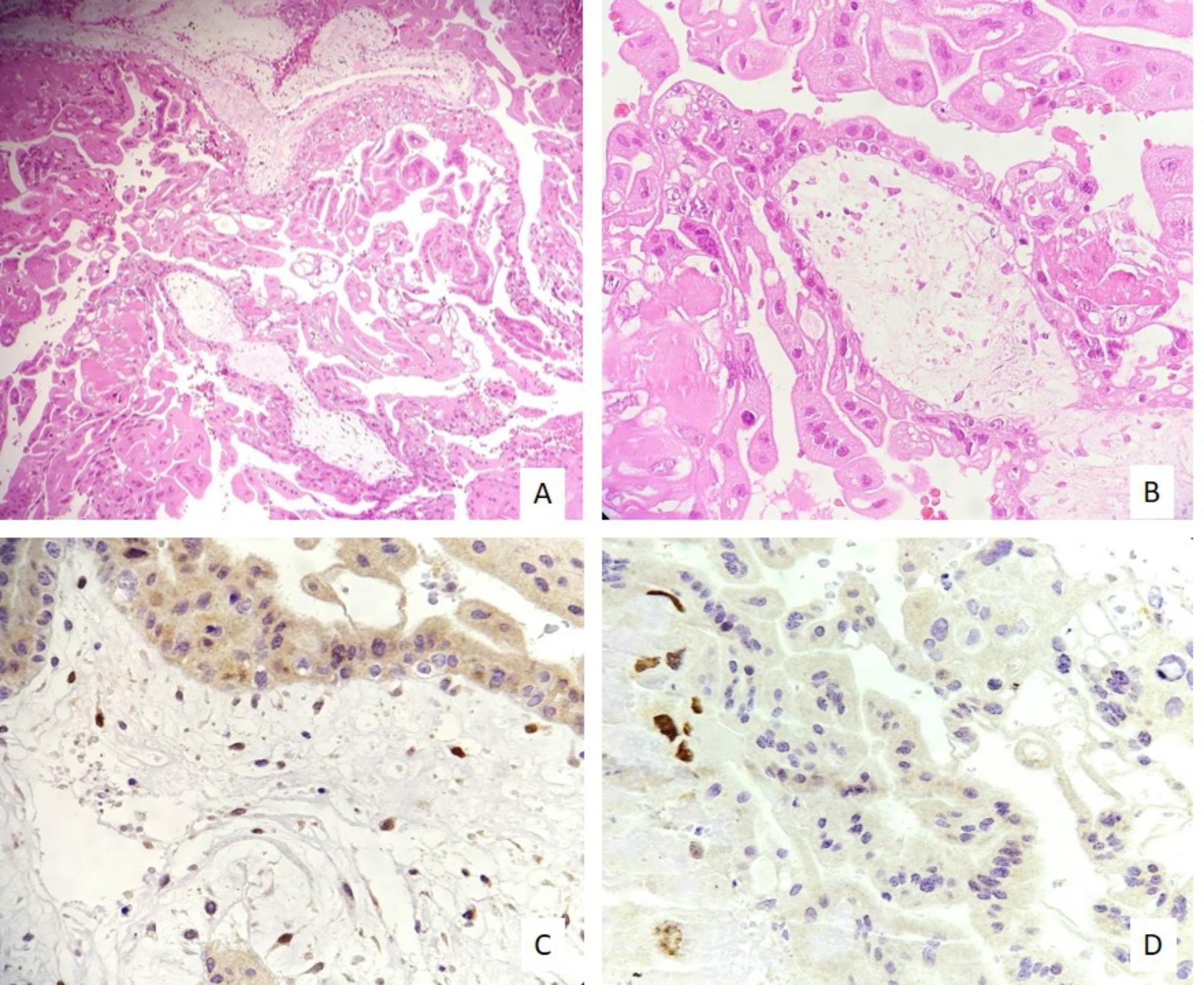
**A, B)** Microscopic examination of complete mole shows hydropic villi with a significant proliferation of trophoblasts (100X, 400X). **C)** Strong positive staining in more than 50% of villous stromal cells and **D)** negative staining in syncytiotrophoblasts (400X)

**Fig. 4:**
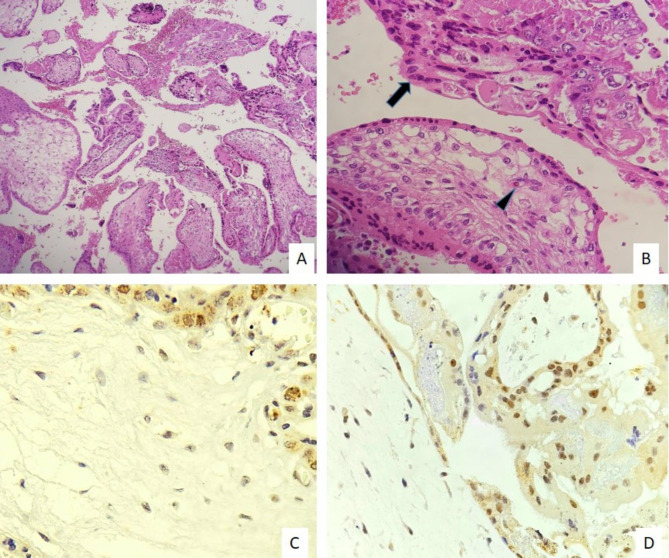
**A, B)** Microscopic examination of partial mole reveal two population of normal villi containing N-RBCs (arrowhead) and hydropic villi with the proliferation of trophoblasts (arrow) (100X, 400X). **C)** Negative/ weak staining of villous stromal cells and **D)** positive staining in syncytiotrophoblasts (400X)

In this study, the expression level of the Twist-1 marker in stromal cells of villi as well as syncytiotrophoblasts was evaluated quantitatively (percentage of positive cells) and qualitatively (staining intensity) separately and as a total comprehensive score. The percentage, intensity, and overall comprehensive scores of Twist-1 expressions were significantly higher in the villi stromal cells in the CM and these values ​​were lower in the syncytiotrophoblasts (p < 0.001) (Figs. 3, 4). Analysis of ROC curves revealed moderate to strong staining in more than 50% of villous stromal cells as the best cut-off value, which can differentiate CM and PM with 89.5% sensitivity and 75% specificity. In syncytiotrophoblasts of CM, negative or weak staining in less than 10% of syncytiotrophoblasts, can distinguish CM and PM with 83% sensitivity and 60% specificity.

There are limited studies about the diagnostic value of Twist-1 for the diagnosis of molar pregnancies. In a study by Rabab A Moussa (2018), they assessed whether the expression of Twist-1, Ki-67, and E-cadherin can guide the differential diagnosis of CM, PM, and hydropic abortion (HA). Differential expression of Twist-1, Ki-67, and E-cadherin was analyzed in gestational products from 55 cases of CM, PM, and HA using immunohistochemistry. Prior to analysis, the studied cases were confirmed by flow cytometric assessment of DNA ploidy and p57 immunostaining. They suggested that a positive stromal score of more than 73 can distinguish CM from PM and HA with 100% sensitivity, 100% specificity, 100% positive predictive value (PPV), and 100% negative predictive value (NPV). In their study, syncytiotrophoblasts in none of the CM cases showed nuclear staining [15].

Although the results of both studies show high diagnostic accuracy of the Twist-1 marker in differentiating the subtypes of molar pregnancy, the cut-off values ​​are different. The sensitivity and specificity of diagnosis in our study are lower than the study of Moussa et al. Since the qualitative values ​​of Twist-1 expression intensity have been used to analyze the results and the scoring of this variable can be different between different observers, this can affect the numerical values ​​of the data and the overall cut-off. The sample size examined in our study was more than the previous study. The potential differences in the efficacy of the antibodies and IHC staining protocols may also influence the results. Unfortunately, in our study, molecular evaluation was not available to confirm the cases with PM diagnosis. Strict morphological criteria and IHC staining for the P57 marker were used to detect molar pregnancies and differentiate complete from PMs. Only those cases that were agreed upon by two expert gynecological pathologists were included. There may be a possibility of Hydropic abortion misdiagnosis with PMs. In spite of that, by using strict morphological criteria and confirmatory p57 IHC staining, we are undoubted about the diagnosis of CM cases.

The results of both studies, in addition to the introduction of a new IHC marker (Twist-1), raise the hypotheses regarding the different pathogenesis of molar diseases. Twist-1 is a transcriptional regulator that plays a role in mesodermal-derived tissues such as the uterus in stem cell differentiation [18, 19]. The role of this molecule in the formation of decidual tissue in the uterus has been suggested in previous studies [19]. On the other hand, the molecule is a negative regulator of cytokine expression that is involved in cell-to-cell adhesion proteins such as E-cadherin and epithelial-mesenchymal transition (EMT). The reduction of E-cadherin expression and the EMT process plays an important role in creating the aggressiveness of trophoblastic cells to penetrate the uterine wall and form the placental tissue [14]. Aberrant expression of E-cadherin in invasive moles compared to non-invasive moles has been suggested in previous studies [14, 20]. Increased expression of Twist-1 in the process of carcinogenesis and the development of EMT properties, resulting in an increased invasive capacity, metastasis, and poor prognosis in various tumors, have been investigated [21–25].

In our study and the study of Moussa et al., a strong and significant increase in the expression of the Twist-1 marker was observed in the stromal cells of villi in CMs compared to the cases of PMs. Risk of invasion and developing choriocarcinoma in CMs is three or four times higher than PMs [1]. Excessive proliferation, atypia and mitosis of trophoblastic cells in CMs is correlated with their more aggressive behavior [26]. The results of our study suggest an additional pathogenic mechanism indicating more invasive nature of the villous stromal cells in CMs. The opposite result was observed in the expression of Twist-1 in the syncytiotrophoblasts. In CMs, this expression was significantly lower than in PMs. The Twist-1 molecule is involved in the fusion of cytotrophoblasts and the formation of syncytiotrophoblasts [20]. Syncytiotrophoblasts play an effective role in the transfer of nutrients, gases, and waste products between the mother and the fetus. Dysregulation of this process has been suggested in pregnancy complications such as pre-eclampsia and IUGR and recurrent pregnancy loss [20, 27–29]. The results of our study are suggestive of disturbance in this process in CMs and a more effective event in PMs.

Despite high sensitivity and specifity of Twist-1, there is no superiority for diagnostic utility, compared with the previously well-known P57 IHC marker. Banet et al. demonstrated high correlation of P57 expression with molecular genotyping results in CMs. P57 is almost always positive in CMs (with more than 99% accuracy). So it’s an extremely reliable, easy to perform or interpret method for the diagnosis of CMs in routine practice [12, 30].

Distinction of PMs and hydropic abortions is a more challenging concern in daily practice of the pathologists. Due to our limitation in selection and confirmation of PMs, we couldn’t address this issue. In the study of Moussa et al. villous stromal cells in PMs expressed more significant percentage of Twist-1. But expression in syncytiotrophoblats was almost similar. Nowadays, molecular genotyping which is an essential factor for the diagnosis of PMs is the most reliable diagnostic method.

## Conclusion

A higher expression of Twist-1 in villous stromal cells of hydatidiform moles is a sensitive and specific marker for the diagnosis of CMs. An elevated expression of this marker in villous stromal cells suggests another pathogenic mechanism for more aggressiveness of CMs in addition to the characteristics of trophoblast cells. The opposite result was obtained in the expression of Twist-1 in the syncytiotrophoblasts, compatible with defects in the process of formation of these supportive cells in CMs.

## Data Availability

The data that support the findings of this study are available from the Hospital information system of Imam Khomeini Hospital affiliated with TUMS but restrictions apply to the availability of these data, which were used under license for the current study and so are not publicly available. Data are however available from the corresponding author (F. N) on reasonable request with permission of the TUMS.

## Abbreviations

HM: Hydatidiform mole
CM: Complete mole
PM: Partial mole
IHC: Immunohistochemistry
GTN: Gestational trophoblastic neoplasm
NMS: Non-molar specimen
EMT: Epithelial-mesenchymal transition
CS: Comprehensive score
ROC curve: Receiver operating characteristic curve
AUC: Area under the curve
PPV: Positive predictive value
NPV: Negative predictive value
GTD: Gestational trophoblastic disease
HA: Hydropic abortion
